# Associations between hand function and electrophysiological measurements in hand osteoarthritis patients of different ages with or without carpal tunnel syndrome

**DOI:** 10.1038/s41598-020-74795-2

**Published:** 2020-11-06

**Authors:** Young Hoon Kim, Eun Young Han, Jinseok Kim, Kyu-Bum Seo, Young Tae Jeon, Sang Hee Im

**Affiliations:** 1 Department of Medicine, Graduate School, Jeju National University, Jeju, Korea; 2 Department of Rehabilitation Medicine, Regional Rheumatoid and Degenerative Arthritis Center, Jeju National University Hospital, Jeju National University College of Medicine, Jeju, Korea; 3 Department of Internal Medicine, Regional Rheumatoid and Degenerative Arthritis Center, Jeju National University Hospital, Jeju National University College of Medicine, Jeju, Korea; 4 Department of Orthopedic Surgery, Regional Rheumatoid and Degenerative Arthritis Center, Jeju National University Hospital, Jeju National University College of Medicine, Jeju, Korea; 5 Department and Research Institute of Rehabilitation Medicine, Severance Hospital, Yonsei University College of Medicine, Seoul, Korea

**Keywords:** Rheumatic diseases, Neuropathic pain

## Abstract

Osteoarthritis is a common degenerative disease that most frequently involves the hand. The objective was to compare clinical functional outcome measures including hand grip, pinch strength, and dexterity with various electrophysiological measures in patients of different ages with hand osteoarthritis with or without the presence of carpal tunnel syndrome (CTS). Patients with hand osteoarthritis (208 patients, 404 hands) who underwent hand-function tests and motor and sensory nerve conduction studies (NCS) between June 2015 and June 2016 were enrolled. The patients’ hands were assigned to carpal tunnel syndrome (CTS) (206 hands; mean age, 56.37 ± 10.52; male:female, 46:160) or control groups (198 hands; mean age, 57.88 ± 9.68; male:female, 55:143). The strength of hand grip and lateral pinch, the time required to complete the nine-hole pegboard test (9HPT), and motor and sensory nerve conduction parameters were measured and compared across age groups and between hands with or without CTS. The CTS group showed significantly lower hand grip and lateral pinch strength, and a longer time to complete the 9HPT in comparison with the control group. Female patients showed significantly lower hand grip and lateral pinch strength than male patients. However, there was no difference in the 9HPT completion time between genders. Multivariate regression analysis identified the amplitude of the median compound muscle action potential (CMAP), age, and male gender as independent predictors of grip strength (adjusted R_2_ = 0.679), and amplitude of median CMAP and male gender as independent predictors of KP strength (adjusted R_2_ = 0.603). Velocity of median CMAP, amplitude of median sensory nerve action potential, and age were identified as independent predictors of 9HPT time (adjusted R_2_ = 0.329). Nerve conduction measurements were significantly related to hand-function test results, and CTS induced significant deficits in strength and performance of the affected hand.

## Introduction

Osteoarthritis is a common degenerative disease that most frequently involves the hand^1^. As the hand is the most commonly used organ, hand osteoarthritis can cause serious disability and functional deficits in the activities of daily living (ADL)^2^. In addition, carpal tunnel syndrome (CTS) is the most common peripheral entrapment neuropathy in the upper extremities^3^. Advanced CTS with thenar muscle atrophy indicates decreased grip and pinch strength^4^, which are related to the upper extremity disability^5^. However, despite several previous studies reporting a causal link between CTS and basal thumb osteoarthritis^6,7^, Shin et al. suggested that basal thumb osteoarthritis was not more prevalent in a CTS group, compares with a non-CTS group, and that the radiographic severity of basal joint arthritis of the thumb was not related to the electrophysiological severity of carpal tunnel syndrome in elderly Korean subjects^8^. Moreover, CTS can result from maladaptive changes around the wrist joint due to repetitive hand use in the ADL.

The electrophysiological severity of CTS is usually determined by complex criteria including both sensory and motor nerve conduction studies (NCS). Hand-intrinsic muscles are innervated by the ulnar nerve and the median nerve, and they are controlled by a delicate mechanism. Hand and finger strength and dexterity are reported to represent various hand performance abilities^9^, and reflect independency in ADL^10^.

Although nerve conduction studies (NCS) are a standard method for diagnosing peripheral neuropathy including CTS, and both high sensitivity and specificity are reported in numerous studies^11^, there remains some controversy over their validity, because other studies have reported relatively high rates of false negative and false-positive tests^12^. These might be caused by the wide range of reference values for both distal sensory latency (DSL) and distal motor latency (DML) for diagnosis of CTS. Khosrawi and Dehghan^11^ suggested a DSL cut-off of 3.6 ms with a sensitivity of 87% and specificity of 91%, and a DML cut-off of 4.2 ms with a sensitivity of 70% and specificity of 100%, and these values have been widely used for the electromyographical diagnosis of CTS in Korean rehabilitation medicine.

The aim of this study was to investigate the objective value of clinical functional outcome measures including hand grip, pinch strength, and dexterity in the presence of CTS and in accordance with age, and to assess possible correlations with various electrophysiological values to provide reference values for clinical functional outcome measures in hand osteoarthritis.

## Methods

### Subjects

This case–control study included 208 patients (404 hands) who visited the outpatient clinic of our institution and underwent an NCS between June 2015 and June 2016. The patients met the clinical criteria for symptomatic hand osteoarthritis proposed by the American College of Rheumatology:^13^ (1) pain, aching, or stiffness of the hands; (2) hard tissue enlargement involving at least two of ten selected joints; (3) swelling of fewer than three metacarpophalangeal joints; (4) hard tissue enlargement of at least two distal interphalangeal joints or deformity of at least one of the ten selected joints.

Subjects with cervical radiculopathy, diabetes mellitus, thyroid disease, peripheral polyneuropathy, connective tissue diseases or joint contracture or deformity were excluded.

CTS was confirmed by clinical symptoms such as pain, numbness, or sensory change along the median nerve distribution, and by electrophysiological diagnosis using American Association of Neuromuscular & Electrodiagnostic Medicine guidelines^14^.

CTS (206 hands; mean age, 56.37 ± 10.52; 46 male, 160 female) and non-CTS groups (198 hands; mean age, 57.88 ± 9.68; 55 male, 143 female) were assigned according to the classifications of the NCS. Medical history, physical and neurological examinations, and *electrophysiological findings* were reviewed retrospectively for all patients.

This study was conducted in accordance with the recommendations of the institutional review board of Jeju National University hospital, which also approved the protocol used in this study. Given the retrospective study design, the need for informed consent was waived. Thus, the institutional review board of Jeju National University Hospital waived the informed consent from patients in this study (ethical approval number: JNUH-IRB 2016-06013).

### Assessment of electrophysiological findings

NCS were performed using a Medelec Synergy electromyography machine (Medelec Synergy, Oxford, UK) with surface electrode recording^15^.

The skin temperature was maintained above 32 °C. The condition of the motor nerves was evaluated for the median nerve innervating the abductor pollicis brevis muscle and the ulnar nerve innervating the abductor digiti minimi muscle. The distance between the distal stimulation site and the active recording electrode was set at 7 cm in the upper extremities. Parameters for distal latency (DML), amplitude, and velocity were adopted on the basis of the compound muscle action potential (CMAP)^15^.

Sensory nerve conduction studies were performed antidromically for the median and ulnar nerves with an active electrode at a position 14 cm above the nerves. At least ten responses were averaged. The latency (DSL) and amplitude of the sensory nerve action potential (SNAP) were obtained. The criteria for diagnosis of CTS were a DSL of 3.6 ms and a DML of 4.2 ms^11^.

### Measurement of hand function

A portable digital hand dynamometer (Jamar dynamometer, Sammons Preston, Bolingbrook, IL, USA) was used to measure the isometric grip strength of the hand (in kg)^16^, while a Jamar pinch gauge (PG60, B&L Engineering, Tustin, CA, USA) was used to measure key pinch strength. The dynamometers were calibrated prior to the first trial. Participants were tested while in a sitting position with the measured arm by their side and not touching the body, and the shoulder adducted and neutral. The elbow was flexed 90°, the forearm neutral, and the wrist extended 0°–30° with ulnar deviation^17^. The participants were asked to squeeze the dynamometer with each hand to facilitate measurement of their grip strength, and to pinch the baseline pinch gauge with the thumb and the lateral aspect of the middle phalanx of the index finger for measurement of key pinch strength^18^. The largest values of all three measurements were recorded.

The wooden nine-hole pegboard test (9-HPT; Jamar, Sammons Preston) was used to measure hand and finger dexterity. The 9-HPT consists of a molded dish situated next to a nine-hole pegboard (31 × 26 × 4 cm) with nine wooden pegs (0.6 cm in diameter). Sitting participants were instructed to place and remove all nine pegs one at a time as quickly as possible at mid-chest level, with the order of placement not being determined^19^. The score was the total time in seconds required to complete the task. The timing began on first contact with a peg and ended with return of the final peg to the dish. The shortest time of two trials for each hand was recorded.

### Statistical analysis

Simple descriptive statistics were used to characterize the samples and distributions of the variables. Data are presented as mean ± SD (standard deviation) for continuous variables, including age, hand-function test results, and all electrophysiological parameters. All clinical and electrophysiological parameters were analyzed using the Wilcoxon paired *t*-test according to the presence of CTS. One-way analysis of variance (one-way ANOVA) was used to compare average values according to age level. The chi-square test was used for categorical variables.

Pearson’s correlation was used to assess the relationships between clinical and electrophysiological parameters. Spearman correlation was used to assess the relationships between non-parametric values.

Multivariate linear regression analysis using a backward selection linear regression model was employed to determine strong predictors amongst the NCS measures for each clinical functional outcome measure. All statistical analysis were performed using the statistical package ver. 20.0 (SPSS Inc, Chicago, IL, USA). A statistically significant difference was considered with a p-value of less than 0.05. Estimation of minimal sample size and statistical power was determined by G* Power program^20^.

## Results

### General characteristics, hand function, and electrophysiological parameters of all patients

The average age of the participants was 57.1 ± 10.1 years, and the majority of the patients were women (71.2%, 148/208). One hundred and one (25.0%) of the recruited hands belonged to male subjects and 303 (75%) to female subjects. Two hundred and five of the total included hands were right hands (50.7%).

The 404 included hands were divided into six groups on the basis of age: 20–29 years (n = 24); 30–39 years (n = 44); 40–49 years (n = 57); 50–59 years (n = 130); 60–69 years (n = 64); and ≥ 70 years (n = 52). The mean values of function test and electrophysiological results are indicated in Table 1 according to age group. All parameters except for the distal latency of the ulnar CMAP showed statistically significant differences across the age groups.

**Table 1:** Parameters of hand functional tests and electrophysiological findings among age groups.

Age group	Grip strength (kg)	KP strength (kg)	9HPT (s)	Median SNAP		Ulnar SNAP		Median CMAP			Ulnar CMAP		
				DL	Amp	DL	Amp	DL	Amp	Vel	DL	Amp	Vel
				(ms)	(uV)	(ms)	(uV)	(ms)	(mV)	(m/s)	(ms)	(mV)	(m/s)
20–29 (n = 24)	32.5 ± 13.4	7.4 ± 2.1	15.2 ± 2.1	3.0 ± 0.2	57.3 ± 16.8	3.1 ± 0.2	36.1 ± 13.7	3.1 ± 0.2	10.8 ± 2.1	58.5 ± 4.0	2.5 ± 0.2	11.7 ± 1.9	65. ± 3.5
30–39 (n = 44)	30.1 ± 7.2	7.2 ± 1.7	16.1 ± 1.9	3.2 ± 0.6	46.5 ± 16.6	3.1 ± 0.2	29.4 ± 9.5	3.2 ± 0.6	10.9 ± 1.8	58.3 ± 3.8	2.4 ± 0.2	10.5 ± 1.8	64. ± 4.1
40–49 (n = 57)	26.1 ± 6.8	6.4 ± 1.8	17.3 ± 3.0	3.2 ± 1.3	32.4 ± 17.9	3.1 ± 0.2	26.3 ± 9.5	3.9 ± 1.4	9.3 ± 3.0	54.0 ± 3.7	2.6 ± 1.1	11.0 ± 2.5	66. ± 6.2
50–59 (n = 130)	22.0 ± 6.8	6.0 ± 1.7	17.8 ± 2.7	3.6 ± 1.6	24.2 ± 17.2	3.1 ± 0.2	23.7 ± 9.7	4.1 ± 1.5	8.7 ± 3.0	52.5 ± 9.4	2.5 ± 0.3	10.3 ± 1.8	63. ± 4.3
60–69 (n = 97)	22.9 ± 7.6	6.5 ± 1.7	19.2 ± 2.8	3.7 ± 1.2	21.5 ± 9.4	3.2 ± 0.2	18.6 ± 5.7	3.7 ± 1.2	8.7 ± 3.0	50.9 ± 11.3	2.6 ± 0.2	10.0 ± 1.6	62. ± 5.0
$\ge$ 70 (n = 52)	18.5 ± 4.8	5.5 ± 1.4	20.7 ± 4.9	3.6 ± 1.1	20.3 ± 9.4	3.3 ± 0.2	16.8 ± 3.7	4.0 ± 1.3	7.8 ± 2.4	51.4 ± 8.7	3.4 ± 4.5	9.2 ± 1.9	62. ± 3.2
p-value	< 0.001**	< 0.001**	< 0.001**	< 0.001**	< 0.001**	< 0.001**	< 0.001**	< 0.001**	< 0.001**	< 0.001**	0.136	< 0.001**	< 0.001**

Values are presented as mean ± standard deviation (SD), ** p < 0.01.

*n* numbers, *KP* key pinch, *9HPT* nine-hole pegboard test, *kg* kilogram, *sec* seconds, *m/s* meter per second, *SNAP* sensory nerve action potentials, *CMAP* compound muscle action potentials, *DL* distal latency, *Amp* amplitude; *Vel* velocity.

In all age groups, the mean grip strength and KP strength were significantly higher in males than in females. There were no significant gender-specific differences in 9HPT (Table 2).

**Table 2:** Parameters of hand functional tests according to gender and age groups.

Age group (M:F)	Grip strength (kg)			KP strength (kg)			9HPT (sec)		
	Male	Female	p-value	Male	Female	p-value	Male	Female	p-value
All (101:303)	33.8 ± 7.9	20.5 ± 5.0	< 0.001**	8.5 ± 1.6	5.6 ± 1.2	< 0.001**	18.2 ± 4.3	18.1 ± 3.0	0.753
20–29 (8:16)	47.8 ± 9.5	24.9 ± 6.8	< 0.001**	9.9 ± 1.1	6.2 ± 1.2	< 0.001**	15.2 ± 2.1	15.0 ± 2.1	0.409
30–39 (18:26)	37.2 ± 3.9	25.1 ± 4.2	< 0.001**	8.8 ± 1.2	6.0 ± 1.0	< 0.001**	16.1 ± 1.9	16.0 ± 2.0	0.804
40–49 (16:41)	33.1 ± 4.8	23.3 ± 4.8	< 0.001**	8.1 ± 1.8	5.8 ± 1.4	< 0.001**	17.3 ± 3.0	17.0 ± 2.4	0.391
50–59 (19:111)	34.3 ± 6.7	19.9 ± 4.1	< 0.001**	8.7 ± 2.1	5.6 ± 1.1	< 0.001**	17.8 ± 2.7	17.6 ± 2.3	0.229
60–69 (30:67)	31.3 ± 6.5	19.1 ± 4.3	< 0.001**	8.3 ± 1.3	5.6 ± 1.1	< 0.001**	19.2 ± 2.8	19.5 ± 3.1	0.108
$\ge$ 70 (10:42)	24.4 ± 4.0	17.0 ± 3.8	< 0.001**	7.5 ± 0.8	5.0 ± 1.1	< 0.001**	20.7 ± 4.9	20.4 ± 3.4	0.687

Values are presented as mean ± standard deviation (SD) or (numbers of male hands: numbers of female hands), ** p < 0.01.

*KP* key pinch, *9HPT* nine-hole pegboard test, *kg* kilogram, *sec* seconds.

### Comparisons of general characteristics, hand function, and electrophysiological parameters between the CTS and control groups

There were no statistically significant differences in age or gender between the CTS and control groups (mean age, 58.1 ± 9.8 vs 56.5 ± 9.2, p = 0.09, CTS and control groups respectively; male gender, 46 vs. 55, p = 0.21).

The parameters of the hand-function tests are compared according to gender and the presence of CTS in Table 3. The average grip and KP strengths of the CTS patients were significantly lower than those of the control group, regardless of gender. The mean time to complete the 9HPT was significantly longer in the CTS patients than in the controls, regardless of gender.

**Table 3:** Comparison of general characteristics and hand function tests between carpal tunnel syndrome group and control group in all patients and both genders.

	All patients			Male			Female		
	CTS (n = 208)	Control (n = 196)	p-value	CTS (n = 42)	Control (n = 59)	p-value	CTS (n = 166)	Control (n = 137)	p-value
Grip strength (kg)	21.4 ± 6.4	26.5 ± 9.1	< 0.001**	29.6 ± 6.0	36.8 ± 7.7	< 0.001**	19.4 ± 4.7	21.9 ± 5.0	< 0.001**
KP strength (kg)	5.9 ± 1.7	6.8 ± 1.8	< 0.001**	8.0 ± 1.8	8.9 ± 1.3	0.006**	5.4 ± 1.2	5.9 ± 1.1	0.001**
9HPT (sec)	19.1 ± 3.7	17.0 ± 2.6	< 0.001**	19.5 ± 5.4	17.2 ± 3.0	0.015*	19.0 ± 3.1	16.9 ± 2.4	< 0.001**

Values are presented as mean ± standard deviation *p < 0.05, **p < 0.01.

*CTS* carpal tunnel syndrome, *n* numbers, *KP* key pinch, *9HPT* nine-hole pegboard test, *kg* kilogram, *sec* seconds.

Distal latency of median SNAP was significantly prolonged in CTS group compared to control group (mean values: 4.4 ± 1.0 vs 3.1 ± 0.2, p < 0.001**) and amplitude of median SNAP was significantly decreased in CTS group compared to control group (mean values: 15.4 ± 19.9 vs 42.6. ± 14.6, p < 0.001**). Distal latency of median CMAP was significantly prolonged in CTS group compared to control group (mean values: 4.6 ± 1.2 vs 3.1 ± 0.3, p < 0.001**), amplitude of median CMAP was significantly decreased in CTS group compared to control group (mean values: 7.9 ± 3.2 vs 10.3 ± 1.9, p < 0.001**) and velocity of median CMAP was significantly slowed in CTS group compared to control group (mean values: 50.4 ± 11.1 vs 56.1 ± 3.8, p < 0.001**). For ulnar SNAP, amplitude was significantly decreased in CTS group compared to control group (mean values: 19.1 ± 6.8 vs 27.4 ± 10.9, p < 0.001**).

### Correlations between general characteristics and hand function and electrophysiological parameters

Grip strength was significantly correlated with age (*r* =  − 0.437), male gender (*r* = 0.647), KP strength (*r* = 0.814), 9HPT (*r* =  − 0.270), amplitude of median SNAP (*r* = 0.270), distal latency (*r* =  − 0.163), amplitude (*r* = 0.387) and velocity (*r* = 0.185) of median CMAP, and amplitude (*r* = 0.247) of ulnar CMAP (p < 0.05*).

KP strength was significantly correlated with age (*r* =  − 0.239), male gender (*r* = 0.643), grip strength (*r* = 0.814), 9HPT time (*r* =  − 0.235), amplitude of median SNAP (*r* = 0.130), amplitude (*r* = 0.383) and velocity (*r* = 0.211) of median CMAP, and amplitude (*r* = 0.166) of ulnar CMAP (p < 0.05*).

9HPT was significantly correlated with age (*r* =  − 0.437), grip strength (*r* =  − 0.270), KP strength (*r* =  − 0.235), amplitude (*r* =  − 0.408) of median SNAP, amplitude (*r* =  − 0.362) and velocity (*r* =  − 404) of median CMAP, distal latency (*r* = 0.225) and amplitude (*r* =  − 0.365) of ulnar SNAP, and amplitude (*r* =  − 0.206) of ulnar CMAP (p < 0.05*).

Multivariate regression analysis identified amplitude of median CMAP, age, and male gender as independent predictors of grip strength (adjusted R_2_ = 0.679, p < 0.001**), and amplitude of median CMAP and male gender as independent predictors of KP strength (adjusted R_2_ = 0.603, p < 0.001**). The velocity of median CMAP (*P* < 0.001**), the amplitude of median SNAP (p = 0037*), and age (p < 0.001**) were identified as independent predictors of 9HPT (adjusted R_2_ = 0.329).

## Discussion

Our study found that electrophysiological parameters, especially those of the median motor nerve, were strong predictors of the results of hand-function tests (hand grip, KP strength, and dexterity) in hand osteoarthritis patients. Furthermore, the participants with CTS showed significantly lower hand grip strength, pinch strength, and steadiness of hand dexterity than those without CTS, findings that are compatible with recent reports^21,22^.

Hand strength plays a crucial role in the performance of ADL, and grip and KP (or lateral pinch) strength are the most common assessments of hand strength^19^. Hand grip strength is an objective indicator used for the setting of treatment goals and the evaluation of intervention outcomes^23^, and is also a strong predictor of muscular strength, nutritional status, disability^24^, and multimorbidity^25^.

The 9-HPT is a reliable test for finger dexterity, the lack of which can have a great impact on a person’s performance in daily activities and independent daily living, and their ability to complete work-related tasks^19^. Lack of finger dexterity is mainly caused by impairments to motor fibers in the median nerve. However, the hand grip and lateral pinch use both intrinsic and extrinsic muscles of the hand, most of which are innervated by the proximal median, ulnar, or radial nerves^26^, and may also be compensated by synergistic muscles such as the flexor digitorum superficialis and flexor digitorum profundus, instead of weakened APB or opponens pollicis muscles. Thus, there is some controversy over the relevance of hand functional tests for assessing the severity of CTS or progress made after surgical decompression^11,21^.

Interestingly, while the distal latency of the median sensory and motor nerve is generally used as a diagnostic criterion for CTS, our results demonstrated that the amplitude of the median CMAP was a strong predictor of hand strength, and that the velocity of the median CMAP and the amplitude of the median SNAP were strong predictors of hand dexterity.

The amplitudes of CMAP and SNAP reflect the summated axons of depolarized motor and sensory nerves, while the velocity of CMAP reflects the velocity of the fastest conducting nerve fibers. Thus, our results confirmed the value of NCS as an objective method for the assessment of hand osteoarthritis, even though accurate functional assessment may not be possible because of severe pain or compensation. In particular, it is noteworthy that the amplitude of the ulnar SNAP was lower in the CTS group. Abnormalities of ulnar sensory nerve conduction in CTS have been observed in various studies, and significant correlation between ulnar nerve abnormalities and the severity of median nerve impairment has been shown^27^.

Additionally, we also demonstrated reference values for comprehensive functional outcome measures according to age and gender in persons with hand osteoarthritis. Hand and finger strength tended to decrease with age, and hand dexterity showed the opposite trend; these results are supported by the reported normal values in healthy Koreans, which are comparable or slightly lower^23,28^.

The nerve conduction measurements performed in this study, especially those on the median nerve, revealed sensitive changes in association with age, with the distal latency of the median CMAP and SNAP increasing with age, but the amplitude of the median CMAP and SNAP, and the ulnar SNAP decreasing with age. Other parameters did not show any statistically significant age-specific differences.

As functional impairment in hand OA may be as severe as in rheumatoid arthritis, assessment and monitoring of the hand function are recommended in the systematic review^29^. In this study, we revealed significant relationship between hand function and electrophysiological findings. In addition, CTS aggravated hand and finger strength and dexterity, findings that were significantly related to objective electrophysiological abnormalities. All functional outcome measures showed statistically significant differences between the patients with and without CTS, with grip strength and KP strength showing lower values in CTS, and the 9HPT test taking longer in those with CTS.

Mean hand grip strength of the dominant hand in men and women was 39.5 and 24.2 kg and the cut-off values for weak hand grip strength were < 28.9 and < 16.8 kg in men and women, respectively in Korean adults^30^. In this study, average hand grip strength in male and female participants were 33.8 kg and 20.5 kg, respectively, which revealed lower hand grip strength than those of average Korean adults but higher than the criteria of sarcopenia. Further studies might evaluate the appropriate criteria of sarcopenia in patients with hand OA.

On the other hand, it is important to identify the functional impairment and sensorimotor deficit in prescribing proper rehabilitation program. Previous studies reported that blood flow restriction training^31^ and tactile discrimination training^32^ improved strength and function and reduced pain and might improve patients’ ADLs and QoL^33^ in patients with hand OA. So, comprehensive rehabilitation program should compose of sensory retraining as well as motor control and further study might be needed is its beneficial effects on patients’ functional status, ADLs and QoL.

To the best of our knowledge, our study is the first to analyze the correlation of hand strength & dexterity with various electrophysiological parameters using a weighted polynomial regression in relatively large number of participants and to compare according to age span, gender, and presence of CTS and to confirm the value of electrophysiological study in patients with hand OA as an objective indicator of hand dysfunction.

There are several limitations to this study. First, the study design was cross-sectional, thus we did not investigate cause-and-effect relationships between the parameters associated with severity or prognosis. Second, the all control group was composed of patients with osteoarthritis, showed deficits of hand function tests, compared with normative values reported in previous study. So further studies should be planned to compare with healthy persons. Third, although our result had strength in that male participants were included, the male:female ratio was 1:3, with a consequently slightly low statistical power. But because CTS and hand OA showed high prevalence of female gender, it might reflect the actual clinical situation of disease. Fourth, a quantitative evaluation of pain severity or osteoarthritis severity was not performed. Finally, the direct effects of OA or CTS on basic or instrumental ADL were not assessed.

In conclusion, the results of our study confirmed a strong relationship between NCS and hand functional outcome measures in osteoarthritis, with CTS inducing significant further deficits in the strength and performance of affected hands. However, although there is considerable evidence that functional impairments limit individuals’ abilities to perform ADL, further longitudinal studies might be warranted to confirm their effect on the progression or prognosis of osteoarthritis or CTS.
