# Author Correction: Associations between hand function and electrophysiological measurements in hand osteoarthritis patients of different ages with or without carpal tunnel syndrome

**DOI:** 10.1038/s41598-021-82090-x

**Published:** 2021-02-01

**Authors:** Young Hoon Kim, Eun Young Han, Jinseok Kim, Kyu‑Bum Seo, Young Tae Jeon, Sang Hee Im

**Affiliations:** 1 Department of Medicine, Graduate School, Jeju National University, Jeju, Korea; 2 Department of Rehabilitation Medicine, Regional Rheumatoid and Degenerative Arthritis Center, Jeju National University Hospital, Jeju National University College of Medicine, Jeju, Korea; 3 Department of Internal Medicine, Regional Rheumatoid and Degenerative Arthritis Center, Jeju National University Hospital, Jeju National University College of Medicine, Jeju, Korea; 4 Department of Orthopedic Surgery, Regional Rheumatoid and Degenerative Arthritis Center, Jeju National University Hospital, Jeju National University College of Medicine, Jeju, Korea; 5 Department and Research Institute of Rehabilitation Medicine, Severance Hospital, Yonsei University College of Medicine, Seoul, Korea

Correction to: *Scientific Reports* https://doi.org/10.1038/s41598-020-74795-2, published online 06 November 2020

This Article contains errors in the Acknowledgements section.

“This work was supported by a research grant from Jeju National University Hospital in 2015. The funders had no role in the study design, data collection and analysis, decision to publish, or preparation of the manuscript. This manuscript was based on first author Kim’s Doctoral thesis.”

should read:

“This work was supported by the 2020 education, research and student guidance grant funded by Jeju National University. The funders had no role in the study design, data collection and analysis, decision to publish, or preparation of the manuscript. This manuscript was based on first author Kim’s Doctoral thesis”.

